# Dr. Squire on the Extraction of the Placenta

**Published:** 1800-05

**Authors:** J. Squire

**Affiliations:** Ely Place


### To Dr. BATTY.

SIR,

PERMIT me to offer ſome remarks on the expulſion and extraction of the placenta after delivery; to which I am induced, from the peruſal of two communications on the ſubject, in

---

* It is not unlikely but her feet might ſometimes ſlip, the effect of which muſt be obvious.

in the 11th and 13th Numbers of your valuable Journal.—The practice therein recommended, is ſo oppoſite to rules laid down by the ableſt and moſt experienced men of the preſent time, and, to my own knowledge, ſo dangerous in its tendency, that I conſider it a duty I owe to ſociety, to prevent as far as may be in my power, the mode of treatment therein deſcribed: I wiſh alſo to impreſs on the the minds of thoſe Gentlemen who have honoured me with their attendance at my lectures, the advantages of time and patience in natural labours, and particularly in the management of the placenta, under the various circumſtances which may occur. It is our duty, when the ſafety, health, and life of the patient may depend on the mode of treatment adopted, that it ſhould be ſuch as reaſon, obſervation, and experience, have proved to be moſt ſafe and ſuccesſful. If from any remarks I ſhall make, the pain and danger of an unneceſſary operation, in a ſingle inſtance, may be prevented, I ſhall feel myſelf amply gratified.

"On the expediency of an early delivery of the placenta," Mr. T. Peck ſays,* "It is ſometimes retained in utero from the following cauſes: the *rupture* of the funis, or the irregular contraction of the uterus. Either of theſe cauſes exiſting, it behoves the practitioner immediately to determine his conduct: I ſay immediately, becauſe, in my opinion, the placenta cannot be too ſpeedily removed after the expulſion of the child." Soon after, he directs us, "if the efforts of nature are not ſufficient to expel it in ten or fifteen minutes, to extract it."

I do not conceive in what way the *rupture* of the funis ſhould cauſe a retention of the placenta; it may indeed, embarraſs an operator unaccuſtomed to extract it, and ſhould be among other more important conſiderations, a caution not to pull with a force, which may endanger its ſeparation. To ſay, "that the placenta cannot be removed too ſpeedily after the expulſion of the child," is an aſſertion in defiance of common obſervation and experience, and a practice recurring to the barbariſm of former times. To wait "no longer than ten or fifteen minutes for the efforts of nature," is a poſition which cannot be too ſtrongly reprobated, unleſs flooding, or other untoward accident, ſhould require the aſſiſtance of art; two hours or longer ſhould be allowed for the purpoſe; more eſpecially when the circulation has been hurried, or when the uterus is not diſpoſed to act. In commenting on Mr. Davies's caſe, Numb. XI. page 6, Mr. Peck obſerves, "that he never would

* Medical and Phyſical Journal, No. XIII. p. 221.

would suffer the smallest portion of the placenta to remain in the uterus, if manual operation would prevent it." It is certainly a desirable circumstance to extract the whole; but infinitely safer to leave a part, if the adhesion be such, as to occasion great difficulty or force in the separation; the consequence of such violence might be flooding, inflammation, fever, and death. Mr. P. proceeds to say, that the exhaustion of the patient is not to be regarded at all in attempting the extraction of the placenta; and, that it is a favourable circumstance for that purpose. On the contrary, I am convinced, that it ought to be dreaded as the harbinger of the patient's death. It should be ever remembered, that in a state of debility from profuse hæmorrhage, the removal of the placenta may be fatal to the woman in a very short time, or she may die in the attempt to remove it: the removal is to be considered as a remedy for a present hæmorrhage, not for one which has already happened.

"Another objection to desisting in such case is, the probability of still greater difficulty from the irregular contraction of the uterus." The apprehension is groundless; as that supposed difficulty will easily be overcome by gradual and gentle efforts in the introduction of the hand. Mr. Davies says,* "I waited a *quarter* of an hour (the patient being considerably exhausted) before I proceeded to deliver the placenta." Mr. D. does not tell us that any hæmorrhage happened during this interval; would it not therefore have been proper to have waited till his patient had recovered from a state of debility, before the funis was separated, in attempting so soon after the birth of the child to extract the placenta? The experiment of soliciting the descent of the placenta by pulling at the funis, not unfrequently occasions a detachment in part; consequently a flooding, and the necessity of introducing the hand to extract it; all which might be avoided by patiently waiting the spontaneous action of the uterus. Mr. D. goes on, "an hæmorrhage of too considerable a nature taking place, to trust it to the natural efforts of the system, I endeavoured to lay hold of the substance, and bring it away; herein I was also foiled." Mr. D. then enters upon his cordial plan; "but finding the hæmorrhage rather alarming, and the patient sinking, I resolved, *in less than an hour*, to make another effort." Such an interval in many instances would be extremely hazardous; and digging into the placenta "by thrusting the fingers into the substance," was an unlikely mode of separating the

* Medical and Physical Journal, Numb. XI. p. 6.

whole; but very likely to increafe the flooding, and induce inflammation and fever, with a train of irremediable evils. Detaching the edge, or grafping the fubftance, by extending the fingers over it, is a more fafe and probable method to effect our purpofe. The exhibition of cordials between the efforts to detach the placenta by manual operation, the hæmorrhage continuing, is a practice in direct oppofition to every idea of reftraining uterine hæmorrhage, and fhould be purfued only when the difcharge ceafes, abating fomewhat of the zeal and perfeverance of adminiftrating "a volatile draught every two hours, brandy and water, wine, &c." Wine, broths, with light nourifhment, in fmall quantities, repeated at proper intervals, will gradually and effectually replenifh the fyftem. To conclude this cafe, we may add, that the fubject of it has been extremely fortunate in her recovery; fortunate indeed! "From her unufually exhaufted ftate, repeated faintings, colliquative fweats, and a fmall pulfe not to be counted." When the circumftances are taken into confideration; the funis ruptured, a profufe hæmorrhage enfuing, an attempt at manual extraction not fucceeding, and the difcharge continuing near an hour before a fecond attempt was thought expedient, and, after all, a part of the placenta left behind; we may indulge hope in the moft defperate fituation, this inftance affording a proof of the ftrength and refources of the human conftitution. I will quote fhortly the opinions of fome late and prefent accredited writers on this fubject, and I have great fatisfaction in adding fuch refpectable authorities.

*Smellie, vol. I. p.* 234, *fect.* 5. "If there is no danger from a flooding, the woman may be allowed to reft a little, in order to recover from the fatigue fhe has undergone; and, that the uterus may, in contracting, have time to fqueeze and feparate the placenta from its inner furface." "I alfo find the mouth of the womb is as eafily dilated fome hours after delivery as at any other time."

*Note from Dr. Hunter's Lectures.* "Whether the placenta comes in a few minutes or an hour, ufe little or no force: when the pains come on and bear down, it comes away entire; better thus, than to ufe force, which may bring on floodings: uterus contracting, forces down the placenta."

*Note from Dr. Harvey's Lectures.* "The placenta will generally come away in an hour. By gently preffing with the hand upon the uterus, we affift the contraction, and the placenta will be readily expelled; by this method we run no rifque: fome have advifed the woman to fneeze or cough, in order to loofen or bring away the placenta; thefe methods are dangerous, as they quicken the circulation, and may bring on flooding:

flooding: others advise to pull down the burden by the navel string; if a portion is strongly adherent to the uterus, we may by this force invert the uterus."

*Denman's Introduction to Midwifery.* Barely to mention the name of this well-known author, would be a sufficient sanction for my purpose; but I shall quote his words. Vol. II. p. 367, "I believe we are at length arrived at a state of practice with regard to the management of the placenta, that will with difficulty be improved; a practice founded on common sense and observation, that the placenta ought to be, and is generally expelled by the action of the uterus, in the same manner as the child; feeling ourselves at liberty, and called upon to assist only when that action is not equal to the purpose, or when dangerous circumstances demand our assistance." Here we have rules of sound practice, delivered in perspicuous and precise language, which cannot be perverted or misunderstood. *Ibid. p.* 370. "The mere debility of the patient, is, therefore, often a reason why we ought to wait without making any attempts to hasten the separation or extraction of the placenta, as an immediate separation, natural or artificial, would be an addition to the danger which she was before in."

*Treatise on the Management of Pregnant and Lying-in Women, by C. White,* chap. v. p. 83.—Extraction of the placenta; "Certain pain and danger must attend the operation, and in almost every case the odds are great but it is totally unnecessary." P. 308, "I have likewise known many misfortunes arise from the manual extraction, when it has been improperly or untimely performed, such as inversions of the uterus, &c."

*Hamilton's Outlines of the Theory and Practice of Midwifery, 3d edit.* p. 217, "The introduction of the hand into the uterus, to separate the adhesion, or assist the expulsion of the after-birth, is not perhaps absolutely necessary in one of several hundred cases, if the previous stages of the labour have been properly managed. However cautiously performed, it occasions a considerable degree of pain. It is cruel and barbarous to employ a painful mode of assistance; and it is criminal to hazard the consequence of violence, where the same end may be obtained by gentle means, perhaps by waiting *an hour or two* extraordinary. In every view, the operation of introducing the hand to remove the placenta should only be employed in the most urgent cases."

*Essays on the Practice of Midwifery, by W. Osborn,* M. D. p. 39, "The natural expulsion of the placenta is both easier and safer than the artificial extraction, however skilfully performed."

*Practical Essays on the Management of Pregnancy and Labour, by John Clarke,* M. D. p. 23, "The hasty delivery of the placenta, immediately after the birth of the child, can never be necessary except in cases of hæmorrhage, and must endanger the life of the woman in many cases, particularly after tedious and lingering labours, where the uterus is indisposed to act."

*Observations on Human and Comparative Parturition, by R. Bland,* M. D. A. S. S. "The detension of the placenta rarely if ever happens, unless when it is diseased, or labour has been hastened, or has commenced prematurely. From what I have been able to observe, or learn from inquiry, this case of retained placenta does not occur so often as once in two hundred labours."

To the concurring testimonies which I have adduced against "the expediency of an early delivery of the placenta," I might bring forward my highly esteemed friends and colleagues in the lying-in charity, who, from their great experience, judgement, and skill, are confessedly competent, in all points of practice, to deliver a decisive opinion; and whose reputation can receive no addition from my praise. Enough, I trust, has been said to seal judgement of the matter in question; and one more important surely cannot be, than the health and safety of those who are given to us to heighten our joys and alleviate our sorrows. I shall, at a future period, enlarge these observations on the management of the placenta, under different circumstances, which will be published in a practical treatise comprehending the different classes of labours, &c. I am,

Sir,

Your most obedient servant,

J. SQUIRE.

*Ely Place,*
*March* 17, 1800.

*P. S.* Since writing the preceding remarks, I have perused, in your Journal, No. XIV. p. 333, Mr. Davies's "elucidation of his case," in answer to Mr. Peck. Both gentlemen are agreed upon an early delivery of the placenta. I have already expressed my opinion upon that point of practice, confirmed by the testimony of gentlemen high in rank in their profession, of acknowledged abilities, and the greatest experience. Mr. Davies takes notice, in this latter communication, of Mr. Peck's having mentioned two causes only of the retained placenta, "the *rupture* of the funis, and the irregular contraction of the uterus." The first is not a cause, but the inaction, or insufficient action, of the uterus, not noticed by either of the gentlemen; it is more frequently a cause, than the irregular action or schirrous adhesion, and here an early delivery would be inexpedient. In continuation of the history of the case, Mr. D. tells tells us, the exhaustion of his patient "was owing to the *fatigue* occasioned by the previous labour, for no material hæmorrhage had taken place at that time." Stronger reasons could not be offered for witholding any attempts to extract the placenta; and I can only repeat my former remark, that in a case so circumstanced, observation and experience instruct us to forbear any interposition, till the recovery of the patient from a state of debility; by which the danger of premature and hasty attempts to deliver the placenta may be avoided. In this, and other instances of operative cases, it would be happy for the subjects of them, that we recollected the maxim, *Naturâ monstrante viam.* When men, who have had no experience, advance erroneous opinions as rules of practice, (and such I conceive the statements brought forward by these gentlemen) they may mislead the ignorant and unwary. I hope and trust, however, that future experience and observation may so far influence their judgement, as to induce them to adopt more rational principles, and a less dangerous practice.

*Ely Place, April* 12, 1800. J. S.

